# Feasibility of [^15^O]H_2_O PET-CT for quantifying lower limb muscle perfusion in peripheral arterial occlusive disease: a pilot study

**DOI:** 10.3389/fnume.2025.1672054

**Published:** 2026-01-02

**Authors:** Goudje L. van Leeuwen, Richte C. L. Schuurmann, Charalampos Tsoumpas, Milah. N. Baumann, Riemer H. J. A. Slart, Jean-Paul. P. M. de Vries

**Affiliations:** 1 Department of Surgery, Division of Vascular Surgery, University Medical Center Groningen, University of Groningen, Groningen, Netherlands; 2 Department of Nuclear Medicine and Molecular Imaging, University Medical Center Groningen, University of Groningen, Groningen, Netherlands; 3 Department of Biomedical Photonic Imaging, Faculty of Science and Technology, University of Twente, Enschede, Netherlands

**Keywords:** [^15^O]H_2_O, perfusion imaging, peripheral arterial disease, positron emission tomography computed tomography, reliability

## Abstract

**Background:**

Assessing muscle tissue perfusion in patients with peripheral arterial occlusive disease (PAOD) is challenging, as conventional techniques such as computed tomography angiography and transcutaneous oxygen pressure (TcPO_2_) do not capture perfusion within deeper muscle compartments, which are clinically relevant because symptoms such as claudication manifest in the muscles. [^15^O]H_2_O positron emission tomography computed tomography (PET-CT) enables quantitative measurement of tissue blood flow but has not yet been systematically applied in PAOD. This exploratory study investigated the feasibility and reproducibility of using long axial field of view (LAFOV) [^15^O]H_2_O PET-CT to quantify calf muscle perfusion in PAOD patients, comparing two VOI selection strategies and generating hypotheses for future studies.

**Methods:**

Patients with Rutherford stage 3–6 PAOD undergoing endovascular revascularization underwent [^15^O]H_2_O PET-CT imaging before and after treatment. Muscle perfusion (K1 in mL/100 cm^3^/min) was calculated using a 1-tissue compartment model and image-derived input functions. VOIs were defined using either full muscle contours or standardized spherical volumes. Ten legs from five scanning sessions (each including pre- and post-treatment imaging) were analyzed twice by two observers. Agreement and variability between methods and observers were assessed.

**Results:**

K_1_ values ranged from 1.54 to 5.22 mL/100 cm^3^/min. Both VOI methods enabled reproducible quantification of calf muscle perfusion. Intrarater intraclass correlation coefficients (ICCs) were ≥0.94 for both methods; interrater ICCs were 0.90 for spherical VOIs and 0.96 for muscle contours. Bland–Altman analysis showed no systematic bias. Muscle contours showed slightly higher reproducibility, likely due to anatomical accuracy.

**Conclusions:**

[^15^O]H_2_O LAFOV PET-CT enables robust quantification of muscle perfusion in PAOD. This method is promising for future studies on treatment response and pathophysiology in vascular disease.

## 1 Introduction

Peripheral arterial occlusive disease (PAOD) is a prevalent and serious condition with a significant global burden. Its severity can be classified using systems such as the Fontaine or Rutherford classification. The Rutherford classification is a standardized framework that categorizes PAOD based on clinical symptoms, ranging from asymptomatic disease (Grade 0) to severe limb ischemia (Grade 6) (1). Accurate assessment of lower limb tissue perfusion in PAOD remains challenging but is essential (2, 3). It enables the evaluation of whether perfusion is sufficient for wound healing and could provide guidance for interventionalists during endovascular procedures to determine whether perfusion has improved. Furthermore, an accurate assessment of lower limb tissue perfusion can help decide a safe amputation level for patients with chronic limb-threatening ischemia without revascularization options.

Advanced imaging methods, including computed tomography (CT) angiography and duplex ultrasonography, are commonly used to visualize arterial anatomy and evaluate blood flow (4, 5). These techniques assess macrovasculature but cannot determine microvasculature or perfusion parameters. Use of various noninvasive methods to assess perfusion, such as transcutaneous oxygen pressure (TcPO_2_) measurements, is increasing (6–8). Most of these techniques measure perfusion at the skin level, but are not able to determine perfusion of deeper tissue; for example, in the muscles. Furthermore, the accuracy of these techniques is variable, depending on various factors, due to the patient or the observer (9).

A proven method to measure muscle blood perfusion is by [^15^O]H_2_O positron emission tomography (PET). This technique also allows the measurement of deeper tissue that is inaccessible with conventional methods such as TcPO_2_ (10–13). Moreover, PET enables the assessment of multiple regions within the leg in a single scan (14).

Lower extremity perfusion imaging using PET requires the administration of a radiotracer and is often complemented by an additional CT scan for attenuation correction. In recent years, various PET imaging techniques have been used to assess lower extremity skeletal muscle blood perfusion, mostly in healthy patients, with [^15^O]H_2_O PET showing particular promise (14). Studies have demonstrated that [^15^O]H_2_O, with its high diffusibility and inertness properties, provides reliable quantification of muscle blood perfusion (10, 11, 13, 15). The inflow of [^15^O]H_2_O in tissues (K_1_) directly corresponds to blood perfusion because it represents the rate of tracer influx into the tissue. The unique characteristics of [^15^O]H_2_O, including its rapid and uniform distribution without binding or metabolism, make it an ideal tracer for perfusion measurements (14). The K₁ value is used as a direct and reliable indicator of perfusion, providing accurate quantification of blood flow in various tissues and organs. After intravenous administration, [^15^O]H_2_O is delivered to the tissue with the blood flow, passively diffuses across the capillary membrane into the extravascular space, and is subsequently washed out at a rate proportional to perfusion, providing a kinetic basis for using K₁ as a flow-dependent parameter.

Despite promising results in healthy legs, validation of [^15^O]H_2_O PET in patients with PAOD is lacking. Furthermore, previous studies have used separate scans for different regions to assess perfusion (13, 16). However, a long axial field of view (LAFOV) PET-CT scan has never been used to measure perfusion in the legs. This approach minimizes variability and potential misalignment associated with multiple separate scans, ensuring more accurate and consistent perfusion measurements across the entire imaging field (17, 18). Unlike static PET, which provides only a single time-averaged snapshot of tracer distribution, dynamic LAFOV PET enables full kinetic modeling of [^15^O]H_2_O by capturing tracer inflow and washout over time, allowing true quantification of perfusion across the entire field of view in a single scan. An essential step in analyzing PET scans is the manual selection of a region of interest (ROI), which defines the area to be evaluated. For perfusion measurements, this ROI is further extended into a volume of interest (VOI), enabling the three-dimensional analysis of the calf muscles. However, this manual selection process may introduce variability between studies, patients' legs, and observers. The aim of this exploratory reproducibility analysis was to assess the intra- and interrater agreement of perfusion measurements using [^15^O]H_2_O PET-CT in calf muscles of patients with PAOD. The secondary aim was to compare two VOI selection methods for the muscles to explore their impact on reproducibility and clinical applicability, with the intention of generating hypotheses for future validation studies.

## 2 Methods

### 2.1 Study population

This exploratory reproducibility pilot study included three (two patients scanned pre and post endovascular treatment, one patient scanned once) patients with PAOD. In each patient, one leg was classified as Rutherford 3–6 and scheduled for endovascular revascularization, whereas the contralateral leg was classified as Rutherford 0-3 and was not scheduled for treatment. Patients were enrolled between September 2023 and July 2024. Exclusion criteria included a history of stenting or bypass surgery in the same vascular trajectory as well as prior amputations.

This pilot study was part of a larger research project investigating the utility of [^15^O]H_2_O PET imaging to detect changes in perfusion after endovascular treatment. This study focuses specifically on the intra- and interrater agreement of perfusion measurements using [^15^O]H_2_O PET-CT in calf muscles of patients with PAOD, and does not include an evaluation of clinical outcomes. The findings are intended to explore reproducibility and serve as a basis for generating hypotheses for future studies, including those that may incorporate clinical outcomes. The study was conducted in accordance with the Declaration of Helsinki, and the study protocol was approved by the University Medical Center Groningen (UMCG) Ethics Committee. All patients gave written informed consent before participating in the study.

### 2.2 [^15^O]H_2_O PET-CT

For the [^15^O]H_2_O PET-CT, a Siemens Biograph Vision Quadra (Siemens Healthineers, Knoxville, TN, USA) long (106-cm) axial fields of view (LAFOV) PET-CT scanner was used. Patients underwent PET-CT scanning twice—before the endovascular intervention and a maximum of 6 weeks after the intervention. To minimize physiological variability, patients refrained from caffeine for 24 h, strenuous activity for 48 h, and smoking after 10:00 PM prior to the scan.

**Figure 1:**
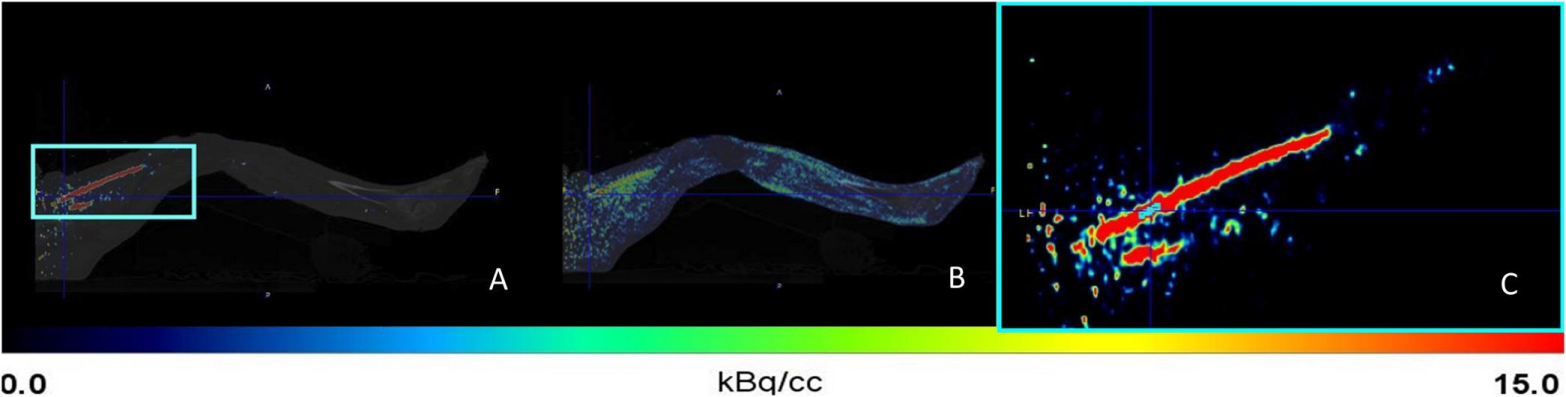
Sagittal planes of the left leg from the first (preintervention) scan of the first patient. **(A)** Fusion of positron emission tomography (PET) and computed tomography (CT) scan at an early time frame (frame 5; 19 s). **(B)** Fusion of PET and CT scan at a late time frame (frame 22; 309 s). The turquoise box in panel A highlights the superficial femoral artery (SFA), located anterior to the adductor magnus muscle and posterior to the vastus medialis, corresponding to the area used for further analysis and shown in greater detail in panel C. Turquoise = region used for arterial time–activity curves.

The protocol for quantifying perfusion was custom-made for the current study but was based on other studies in the literature (16, 19) and clinical protocols at UMCG. The protocol consisted of a low-dose CT (LDCT) scan, followed by a 6 min dynamic PET acquisition, covering the area from the feet up to the abdomen, with the entire pelvis in the FOV. The legs were secured in a fixed position using a bandage and pillows to prevent movement without applying constriction because this could potentially influence the perfusion measurements. Simultaneously with the initiation of each PET acquisition, participants were administered a standardized 400 MBq intravenous bolus injection of [^15^O]H_2_O using a MEDRAD infusion pump (Bayer HealthCare LLC, Whippany, NJ, USA) starting with a 10-s preflush (0.8 mL/s for 10 s), followed by the [^15^O]H_2_O bolus (8.0 mL/s for 40 mL). In addition, patients underwent whole-body LDCT (with an x-ray tube current of 35 mAs, a tube voltage of 100 kV, and a spiral pitch factor of 1.1) for anatomic information and PET attenuation correction. Fused images of a PET and CT scan are shown in Figure 1.

### 2.3 Data analysis

Image processing and pharmacokinetic analysis of [^15^O]H_2_O were performed with PMOD 4.105 software (PMOD Technologies GmbH, Zurich, Switzerland). The PET list-mode data were reconstructed using the 3D Ordered Subset Expectation Maximization (OSEM) algorithm (4 iterations and 5 subsets), point spread function correction and time-of-flight, and reconstructed into 22 time frames (8 × 5, 3 × 10, 4 × 15, 6 × 30, and 1 × 60 s; ∑ = 370 s). Data were corrected for attenuation, scatter, and radioactivity decay. However, no partial volume correction (PVC) was applied, as its impact was considered minimal in the context of this study. This resulted in images with a matrix of 1.65 mm × 1.65 mm × 1.5 mm voxels. A 1-tissue compartment model (1TCM) was used to calculate the K1 rates. The 1TCM assumes tracer exchange between blood plasma and tissue, with influx (K1) and efflux (K2) rate constants. Given the high extraction fraction of [^15^O]H_2_O in well-perfused tissues like skeletal muscle, the blood volume fraction was not explicitly included. The tracer was assumed to distribute homogeneously without further binding or metabolism, making 1TCM suitable for perfusion measurement. Because [^15^O]H_2_O behaves as a freely diffusible tracer without a second kinetically distinct tissue pool, more complex models such as 2-tissue compartment approaches are neither physiologically required nor numerically advantageous.

**Figure 2:**
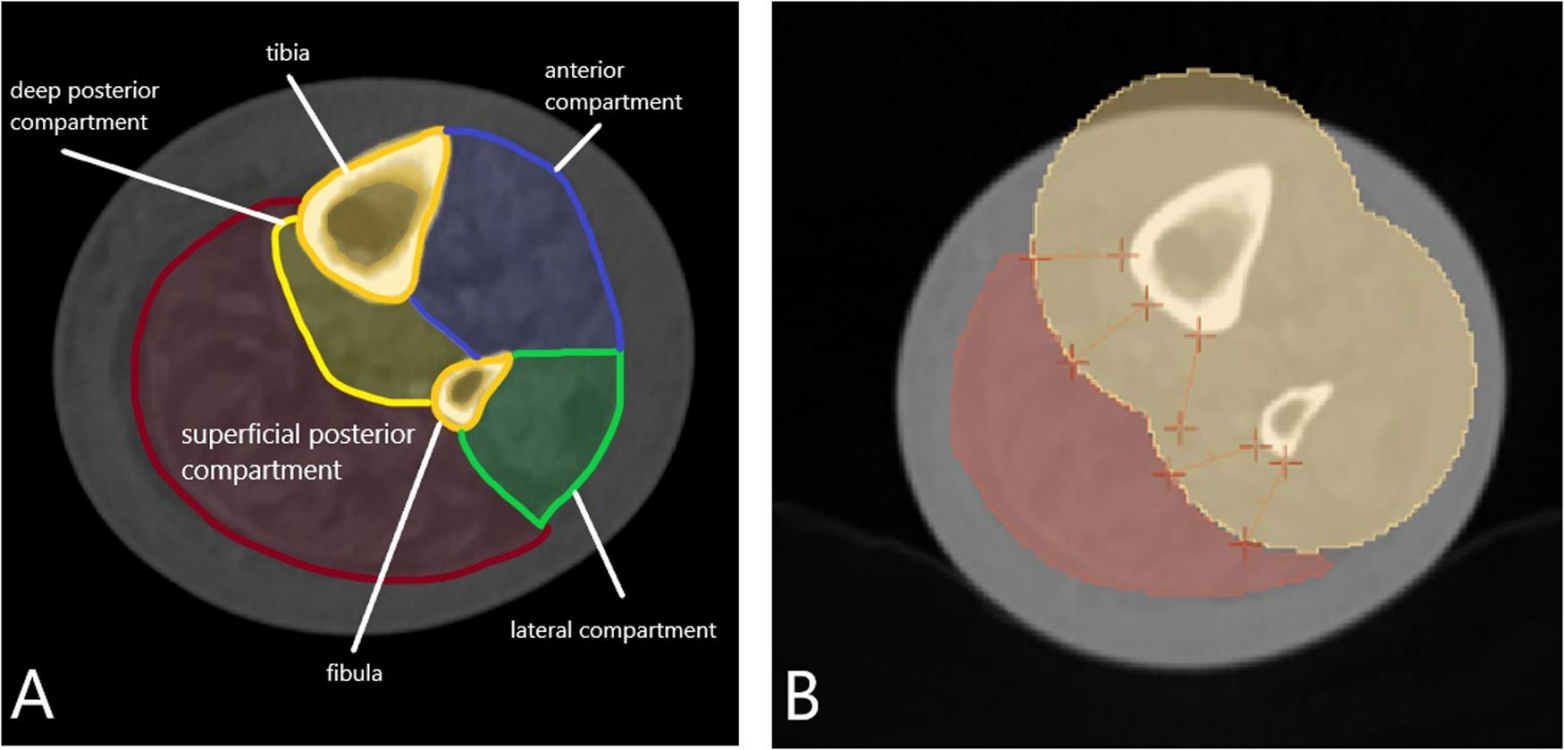
Left (affected) leg from the first (preintervention) scan of the first patient. The compartments of the lower leg are visualized at 60% of its length, measured from the distal end of the medial malleolus to the knee joint space, using low-dose CT. **(A)** The superficial posterior compartment—containing the gastrocnemius and soleus muscles—is defined as the region of interest (ROI). **(B)** Tibial and fibular bones are shown in **yellow**, surrounded by a **20-mm safety margin** to correct for potential patient motion. The **red** area indicates the calf muscles of the superficial posterior compartment. All muscle tissue **posterior (dorsal)** to the bones, including the margin, was delineated as the volume of interest (VOI) for the muscle contour VOI. Yellow = bones + margin; red = muscle compartment.

First, muscle segmentations were performed. The LDCT was loaded into 3D Slicer 4.11 (https://www.slicer.org) and the muscle compartments were identified (Figure 2A). This was done at 60% of the lower leg length, measured from the distal end of the medial malleolus to the knee joint space, based on the findings from the study by Ma et al. (20). Their study demonstrated that cross-sectional single-slice measurements at 60% of the tibial length are most representative of muscle volume and can accurately substitute complete volume segmentations. The calf muscles are commonly involved in claudication symptoms in patients with PAOD, making this region relevant for investigation. Previous studies have examined similar regions of the leg (10, 11).

Due to the limited resolution of LDCT, the soleus and gastrocnemius muscles could not be delineated separately; therefore, the entire superficial posterior compartment was selected as the ROI. To standardize delineation and account for patient motion, a 20-mm exclusion zone was applied around the tibia and fibula (Figure 2B). The ROI was manually segmented in ten 1.5-mm axial slices around the 60% level to create the VOI. LDCT segmentations were then exported and coregistered with the PET scan.

### 2.4 Input functions and VOI-based quantification

Image-derived input functions (IDIFs) were obtained from the bilateral superficial femoral artery (SFA) from the same PET-CT scan. The SFA was chosen because it has a relatively large diameter and provides reliable blood flow even in most patients with PAOD, making it suitable for IDIF extraction. The protocol was based on a previous studies conducted at UMCG, and prior research has demonstrated that the femoral artery is a suitable location for obtaining an IDIF and even smaller arteries are suitable (16, 21). IDIFs were obtained by manually selecting the four highest-intensity voxels as a VOI over 10 consecutive planes in axial direction in the SFA. The first axial plane was positioned 3 cm distally from the bifurcation of the common femoral artery into the SFA and the profunda femoris artery to standardize the selected anatomical location. Other studies have similarly demonstrated the reliable use of IDIFs (21, 22). The VOIs were obtained using a time frame during the first pass of the bolus where the blood pool in the SFA was best visible. After that, the VOI was applied to all other time frames to obtain an IDIF. This process was performed independently for the right and left leg. The VOI is shown in Figure 1C.

**Figure 3:**
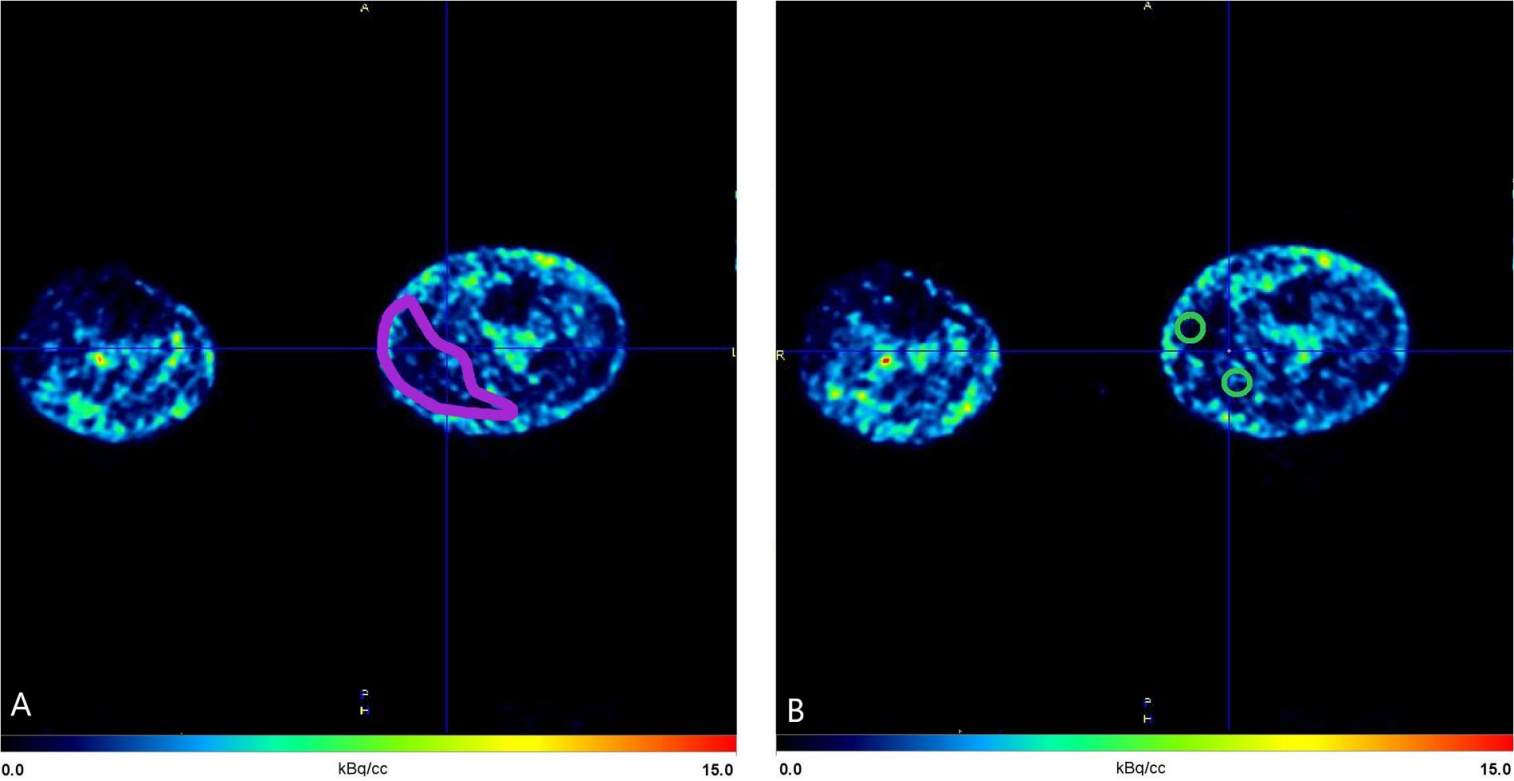
Axial view of the left calf from the first (preintervention) scan of the first patient at time frame 22 (309 s). **(A)** Volume of interest (VOI) of the muscle contour (red). **(B)** Volumes of interest of two combined spherical regions (green), each with a 7.5-mm radius, placed within the superficial posterior compartment. Anatomical orientation: anterior (top), posterior (bottom), medial (right), lateral (left). Red = muscle contour VOI; green = spherical VOIs used for quantitative analysis.

For quantification of perfusion, the segmented calf muscles in the superficial posterior compartment were used. Two types of VOI were used to investigate whether one of the methods was more reliable. The first was a segmentation of the muscle contour, which was the compartment segmentation made based on CT images (Figure 3A). The volumes of these VOIs were patient specific, due to the volume of the muscles. Additionally, the other VOI consisted of two spheres, each with a radius of 7.5 mm, which were placed at the medial and lateral sides of the muscle at the same anatomical location as the previously described muscle segmentation (Figure 3B). These spheres were then combined for perfusion quantification, forming a total volume of 3540 mm^3^ (3.5 mL).

To obtain a time-activity curve, the IDIF from the SFA served as an input function representing arterial blood flow over time (Figure 1C), whereas VOI-based quantification from the muscle contour (Figure 3A) or the spheres (Figure 3B) provided tissue-specific activity. Temporal uptake of the tracer within the VOIs was corrected for volume and plotted. This was combined with the IDIF, used as a reference for input activity, to generate time-activity curves (Supplementary Appendix B). These time-activity curves, extracted from the VOIs, were then used to model muscle blood perfusion dynamics across each time frame using kinetic modelling. As a result of this kinetic modeling, the rate of tracer uptake (K_1_) was determined, representing the perfusion of the segmented calf muscle tissue. K_1_ was calculated as mL/cm^3^/min; however, K_1_ values were converted to mL/100 cm^3^/min for improved readability and ease of interpretation. The blood delay between the superficial femoral artery and the muscle tissue was accounted for in the analysis. This delay was estimated as part of the modeling process and incorporated into the kinetic analysis. Correcting for this delay was essential to accurately align IDIF with the tissue TAC, ensuring that the timing of tracer delivery to the tissue was correctly represented.

To assess intra- and interrater agreement, all measurements were executed twice by two raters (G.v.L. and M.B.). Measurements in one session by one researcher consisted of muscle segmentation, selection of the IDIF, and selection of both types of VOI for each leg, respectively. The sequence of the measurements was randomized before being conducted. There was a 1-week interval between the first and second set of measurements for each researcher. All measurements were completed within a 3-week period.

### 2.5 Statistical analysis

Statistical analysis was performed using SPSS 28 software (IBM Corporation, Armonk, NY, USA). K_1_ values were analyzed as a mean with 95% confidence intervals (CIs). Intra- and interobserver agreement was evaluated using intraclass correlation coefficients (ICCs) based on absolute agreement, calculated with a two-way random effects model for single measures. This model accounts for variability between raters and sessions, providing an estimate of the reliability of individual measurements rather than averaged scores. ICC values below 0.5 indicated poor reliability, values between 0.5 and 0.75 indicated moderate reliability, values from 0.75 to 0.9 were considered good reliability, and values above 0.9 were interpreted as excellent reliability (23). Measurement error for intra- and interobserver agreement was assessed using the limits of agreement and Bland-Altman plots. The limits of agreement were calculated as the mean difference between two measurements ±1.96 times the standard deviation of the mean difference.

## 3 Results

### 3.1 Patients

Three patients were included in the study (Table 1). Two patients underwent the scan before and after endovascular intervention, and one patient only underwent PET scanning before endovascular treatment. All three patients had Rutherford 3–6 in the leg for which they were scheduled for endovascular intervention. Their contralateral leg was classified as Rutherford 0-3. A total of 10 legs were included in the current analysis.

**Table 1:** Clinical characteristics, diagnostic findings, interventions, and outcomes of patients undergoing endovascular treatment for peripheral arterial occlusive disease (PAOD).

Patient & side	Age	Gender	BMI	Ethnicity	ABI (pre/post)	Rutherford (pre/post)	Key CTA findings (pre)	Intervention	Outcome
1 Left	80	Male	26.6	Caucasian	L: 0.49 → 0.60, R: 0.89 → 0.47	L: 5 → 2–3.	L: Stenosis of the SFA and PA, stenoses of PA, occlusion ATA and PTA. R: Stenosis SFA and PA, occlusion ATA and PTA.	BA with stent placement in the SFA, PTA of the TPT, and PA.	Uncomplicated recovery, but due to contralateral complaints, no improvement of walking.
2 Right	77	Male	34.3	Caucasian	R:? → 0.88, L:? → 1.07	R: 2–3 → 2.	R: Occlusion IIA and stenoses SFA, PA, and ATA. L: Stenoses IIA, SFA, and ATA.	Shockwave angioplasty of the SFA, no stent placement required.	Uneventful recovery with improvement in symptoms.
3* Left	66	Female	24.1	Caucasian	L: 0.46 → 0.96, Right: 0.97 → 1.04	L: 2–3 → 1.	L: Occlusion EIA.	BA and stent placement in the external iliac artery.	Uncomplicated recovery with improvement in symptoms.

ABI, Ankle-Brachial Index; ATA, Anterior Tibial Artery; BA, Balloon Angioplasty; BMI, Body Mass Index; CTA, Computed Tomography Angiography; EIA, External Iliac Artery; IIA, Internal Iliac Artery; PA, Popliteal Artery; PTA, Posterior Tibial Artery; SFA, Superficial Femoral Artery; TPT, Tibioperoneal Trunk; L, left; R, right.

### 3.2 Perfusion rates

For each leg, perfusion values in the superficial posterior compartment at 60% of the lower leg were obtained twice, and all measurements were done with the muscle contour-based and spheres VOI. Perfusion values are shown in Supplementary Appendix A and averages ranged from 1.54 to 5.22 mL/100 cm^3^/min. Muscle contour-based perfusion values for the three diseased legs preintervention were a mean of 3.75 mL/100 cm^3^/min (95% CI: 2.96–4.53). Muscle contour-based perfusion values of the two treated legs were a mean of 2.09 mL/100 cm^3^/min (95% CI: 1.87–2.31). Muscle contour-based perfusion values for the five nontreated legs (both before and after intervention) were a mean of 3.05 mL/100 cm^3^/min (95% CI: 2.60–3.49).

Sphere-based perfusion values for the three diseased legs preintervention were a mean of 3.60 mL/100 cm^3^/min (95% CI: 2.96–4.24). Sphere based perfusion values of the two treated legs were a mean of 1.85 mL/100 cm^3^/min (95% CI: 1.31–2.40). Sphere based perfusion values for the five nontreated legs (both pre- and postintervention) were a mean of 3.17 mL/100 cm^3^/min (95% CI: 2.71–3.63).

### 3.3 Intraobserver agreement

The muscle contour-based ICC was 0.89 (95% CI: 0.62–0.97) for observer 1 and 0.96 (95% CI: 0.87–0.99) for observer 2. The sphere-based ICC was 0.91 (95% CI: 0.68–0.98) for observer 1 and 0.94 (95% CI: 0.76–0.99) for observer 2. Bland-Altman plots for intraobserver agreement are shown in Supplementary Appendix C. All ICC values for intraobserver agreement demonstrated excellent reliability, reflecting a strong consistency across repeated measurements by the same observer.

### 3.4 Interobserver agreement

The muscle contour-based ICC ranged between 0.78 (95% CI: 0.37–0.94) and 0.93 (95% CI: 0.74–0.98). The sphere-based ICC ranged between 0.79 (95% CI: 0.39–0.94) and 0.82 (95% CI: 0.45–0.95). Bland-Altman plots for interobserver agreement are shown in Supplementary Appendix C. All ICC values for interobserver agreement demonstrated good to excellent reliability, reflecting a strong consistency between measurements by the different observers.

## 4 Discussion

This feasibility study indicates that perfusion measurements in the calf muscles of patients with PAOD using [^15^O]H_2_O PET-CT are reproducible. We found good to excellent intra- and interobserver agreement for measurements in the superficial posterior compartment at 60% of the lower leg. These findings suggest that this imaging technique, with a standardized scanning and analysis protocol, may provide consistent and reliable results. The absence of systematic bias in Bland-Altman analyses further supports the robustness of the measurements. Importantly, both VOI selection methods evaluated in this study yielded comparable levels of agreement, indicating that either approach can be used in a reproducible manner.

Perfusion values in this study ranged from 1.54 to 5.22 mL/100 cm^3^/min. Several other studies have been conducted on perfusion of skeletal muscles of healthy and ischemic legs using [^15^O]H_2_O and have comparable perfusion values (10, 11, 15). A recent study from Christensen et al. (13) demonstrated that lower extremity skeletal muscle blood perfusion could be accurately measured by [^15^O]H_2_O PET-CT in healthy volunteers. The researchers included 10 healthy volunteers who underwent PET-CT with 400 MBq [^15^O]H_2_O twice on the same day with IDIFs obtained from a separate scan of the heart. VOIs were manually drawn in multiple muscle groups of the lower leg. They then performed kinetic modeling with the 1-tissue compartment model method, and results showed that K_1_ values in healthy legs varied from 1.18 to 5.21 mL/100 cm^3^/min (median, 2.13; interquartile range, 0.87).

All patients reported clinical improvement of their PAOD symptoms, which was also supported by an increased ankle-brachial index (ABI) after revascularization. However, this improvement was not consistently reflected in the measured muscle perfusion values. Several explanations may account for this discrepancy. First, it is possible that muscle perfusion, as measured with [^15^O]H_2_O PET-CT, does not necessarily correlate with patient-reported symptom improvement or ABI changes, suggesting that the initial hypothesis may not hold true. Reasons for this could include microvascular damage, delayed recovery after revascularization, heterogeneous muscle blood flow, or other mechanisms that are not yet fully understood. Nevertheless, if this technique proves to be reliable, whole-limb perfusion imaging could offer substantial clinical value. For instance, it may allow assessment of angiosome-specific perfusion or help identify the optimal amputation level by determining whether local perfusion is sufficient to support primary wound healing. Studies using other perfusion imaging modalities, have shown that perfusion dynamics following revascularization can be associated with clinical outcomes, underscoring the potential usefulness of advanced perfusion measurements in guiding patient management (24, 25).

Certain factors may have affected the PET-CT measurements, despite efforts to minimize their influence. Motion during the scan and physical exertion prior to imaging could have impacted image quality and tracer distribution. In addition, post-acquisition factors—such as inconsistencies in the delineation of the IDIFs or muscle contours—may have introduced variability in the quantitative analysis, even though standardized protocols were used wherever possible. Further research is needed to better understand the relationship between macrovascular and microvascular changes, as macrovascular improvements may not directly reflect microvascular perfusion as measured by PET.

The IDIFs derived from the SFA are not always stable, likely due to slight patient movement. The SFA location is determined using an early time frame in combination with the LDCT images. However, minor patient movements may result in the VOI being partially positioned outside the SFA, which can affect the accuracy of the derived IDIF. Consequently, while the SFA-derived IDIF approach may offer consistency, its reliability and validity compared to other methods, such as the dual-scan approach, remain uncertain and require further investigation. For future studies, full motion correction is essential to maximize quantification accuracy and precision, potentially leading to increased statistical significance.

The current study used two types of VOI selection: muscle contour- and sphere-based VOIs. ICC values were slightly higher for muscle contour-based VOIs; however, both types of VOIs had good to excellent agreement. The muscle contour-based VOI involves segmenting the calf muscle in the axial plane, resulting in a larger VOI that may allow for greater variability in tracer distribution due to its larger size, which could potentially reduce noise and improve reliability. On the other hand, their placement can introduce variability depending on the selected location, potentially affecting reliability. For untrained researchers or future studies, using the sphere-based VOI may prove to be more reliable due to its simplicity and ease of application. However, other shapes and methods may also be viable, and further research into this would be beneficial.

Unlike previous studies measuring perfusion with [^15^O]H_2_O PET-CT, the present study utilizes a long axial field of view (LAFOV) PET-CT. For the advancement of research in patients with PAOD and the measurement of perfusion using [^15^O]H_2_O PET-CT, it is crucial to determine whether the LAFOV scanner provides results comparable to those obtained with separate scans. An advantage of this LAFOV PET-CT compared with other scanners is its ability to simultaneously image the large vessels, such as the SFA, and the calf region, in a single scan. Recent advancements in PET technology, particularly with LAFOV scanners, have demonstrated the potential for significantly reduced radiotracer doses due to their increased sensitivity and resolution. These developments enable shorter acquisition times and lower radiation exposure while maintaining high image quality, making them highly suitable for future applications in perfusion imaging. This integrated approach enhances the accuracy of perfusion measurements by providing consistent anatomical and functional data across the entire scan range, reducing variability and potential misalignment associated with multiple separate scans (17, 18). The SFA was considered the most suitable artery for IDIF determination in this study, because the aorta was outside the field of view. Furthermore, in patients with PAOD, atherosclerotic involvement of the inflow arteries, including the iliac vessels, should be considered. Further research is therefore warranted to evaluate the feasibility and accuracy of aorto-iliac IDIF determination in this patient population.

Using [^15^O]H_2_O PET as quantification for perfusion has several drawbacks. First, cyclotron production and infusion of [^15^O]H_2_O is challenging due to its short half-life. Other radiotracers, such as ^13^N-amonnia and ^82^Rb, may also have potential for assessing muscle blood flow in PAOD research, but [^15^O]H_2_O remains preferable due to its optimal properties for perfusion measurements under both normal and ischemic conditions (14), but a cyclotron on location is necessary (26).

Second, patients are required not to move during the scan, as any patient movement adversely affects the quality and interpretability of PET-CT images (19). This requirement can be particularly challenging for patients with PAOD. However, the short half-life of ^15^O provides an advantageous factor in this context, because the brief scanning duration requires patients to stay still for only a few minutes, in contrast to longer imaging times associated with other tracers with a longer half-life.

### 4.1 Limitations

This study has several limitations. First, the sample size was small, with only 10 legs included in the analysis. While the results demonstrated high reliability, it remains uncertain whether a larger sample size would further enhance the robustness of the findings.

Second, although two types of VOIs were evaluated, we cannot definitively conclude that the selected methods are optimal; however, both methods are common in literature (27) and warranted to be compared. Further studies exploring alternative VOI techniques may provide insights into whether other approaches yield comparable or superior results.

Third, despite the use of strict inclusion criteria, distinguishing between healthy and diseased legs in patients with PAOD remains challenging because this condition is a systemic atherosclerotic disease. Over time, the disease progresses, and a leg initially classified as healthy may no longer remain unaffected. The perfusion values observed before and after the intervention, as well as those compared with the unaffected leg, appear inconsistent and somewhat random. However, given the limited data available, no reliable comparisons can be made between the treated and untreated legs. As such, this was not the aim of the study, and no meaningful conclusions can be drawn regarding the significance of these perfusion values. Although [^15^O]H_2_O PET-CT is already utilized in clinical practice for cardiology (28), its application in PAOD remains a distant prospect.

### 4.2 Future perspectives

LAFOV scanners are promising tools for vascular imaging, but further research is needed. One of the potential applications of quantifying the perfusion of the entire leg, is to assist in selecting the optimal level in the decision of a major amputation. Another important application is the impact of extensive revascularization procedures on the perfusion around ischemic ulcers. These clinical applications require well designed and powered clinical studies to assess the findings. An important next step for future research would be to investigate the relationship between PET-derived perfusion measurements and clinical outcomes like ulcer healing in patients with chronic limb threatening ischemia.

To build on the promising reproducibility of [^15^O]H_2_O PET-CT for assessing muscle perfusion in PAOD, future studies should focus on correlating perfusion changes post-revascularization with clinical outcomes, such as TcPO2 and/or ABI in larger cohorts. A comparison of pre- and post-intervention perfusion values alongside TcPO2 measurements will help clarify the clinical relevance of these imaging results. Based on preliminary power analysis, at least 10–12 patients would be required to detect significant changes in perfusion post-revascularization using PET-CT, while around 16 patients would be needed for TcPO2 measurements to detect a 20% improvement with 80% power at a significance level of 0.05.

In conclusion, this feasibility study suggests that [^15^O]H_2_O PET-CT allows for reproducible perfusion measurements in the lower leg muscles of patients with PAOD, using either muscle contour- or sphere-based VOIs. While the results are promising, the technique has practical limitations, including the need for on-site cyclotron production and vulnerability to patient motion. Further research is needed to confirm clinical applicability and to optimize protocols for broader use.

## Data Availability

The raw data supporting the conclusions of this article will be made available by the authors, without undue reservation.
