# Supplementary material for: Feasibility of [15O]H2O PET-CT for quantifying lower limb muscle perfusion in peripheral arterial occlusive disease: a pilot study
Source: Front Nucl Med. 2026 Jan 2;5:1672054. doi: 10.3389/fnume.2025.1672054 (PMC12808382; doi:10.3389/fnume.2025.1672054)
Supplement: Supplementary file 1 [file Table1.docx]

Supplementary Material

# Appendix A

All K_1_ values.

**Table A1.** Resting perfusion, K_1_, (mL/100 cm^3^/min) in the calf muscle of the first measurement session performed by observer 1.

| Patient | Scan | Left contour | Left spheres | Right contour | Right spheres |
| --- | --- | --- | --- | --- | --- |
| 1 | Preintervention | **3.80** | **3.07** | 4.39 | 4.67 |
|  | Postintervention | **1.75** | **1.21** | 3.30 | 3.36 |
| 2 | Preintervention | 2.11 | 2.08 | **2.33** | **2.82** |
|  | Postintervention | 2.44 | 3.34 | **2.00** | **2.34** |
| 3 | Preintervention | **5.72** | **4.92** | 3.69 | 4.36 |

Bold values indicate the affected leg with Rutherford stages 3-6.

**Table A2.** Resting perfusion, K_1_, (mL/100 cm^3^/min) in the calf muscle of the second measurement session performed by observer 1.

| Patient | Scan | Left contour | Left spheres | Right contour | Right spheres |
| --- | --- | --- | --- | --- | --- |
| 1 | Preintervention | **4.00** | **3.97** | 4.20 | 4.04 |
|  | Postintervention | **2.36** | **1.54** | 2.60 | 2.86 |
| 2 | Preintervention | 2.20 | 2.53 | **2.24** | **2.21** |
|  | Postintervention | 2.37 | 3.01 | **2.08** | **1.97** |
| 3 | Preintervention | **4.87** | **5.06** | 4.92 | 4.07 |

Bold values indicate the affected leg with Rutherford stages 3-6.

**Table A3.** Resting perfusion, K_1_, (mL/100 cm^3^/min) in the calf muscle of the first measurement session performed by observer 2.

| Patient | Scan | Left contour | Left spheres | Right contour | Right spheres |
| --- | --- | --- | --- | --- | --- |
| 1 | Preintervention | **3.44** | **2.73** | 4.58 | 4.88 |
|  | Postintervention | **1.89** | **1.43** | 2.27 | 2.15 |
| 2 | Preintervention | 2.46 | 2.79 | **2.52** | **3.11** |
|  | Postintervention | 2.42 | 2.68 | **2.15** | **1.80** |
| 3 | Preintervention | **5.34** | **4.62** | 2.94 | 3.01 |

Bold values indicate the affected leg with Rutherford stages 3-6.

**Table A4.** Resting perfusion, K_1_, (mL/100 cm^3^/min) in the calf muscle of the first measurement session performed by observer 2.

| Patient | Scan | Left contour | Left spheres | Right contour | Right spheres |
| --- | --- | --- | --- | --- | --- |
| 1 | Preintervention | **3.18** | **3.20** | 4.41 | 4.96 |
|  | Postintervention | **2.55** | **1.99** | 2.26 | 2.43 |
| 2 | Preintervention | 2.14 | 2.03 | **2.58** | **2.75** |
|  | Postintervention | 2.37 | 2.62 | **1.91** | **1.56** |
| 3 | Preintervention | **4.97** | **4.78** | 2.86 | 2.68 |

Bold values indicate the affected leg with Rutherford stages 3-6.
