# Supplementary material for: Feasibility of [15O]H2O PET-CT for quantifying lower limb muscle perfusion in peripheral arterial occlusive disease: a pilot study
Source: Front Nucl Med. 2026 Jan 2;5:1672054. doi: 10.3389/fnume.2025.1672054 (PMC12808382; doi:10.3389/fnume.2025.1672054)
Supplement: Supplementary file 2 [file Table2.docx]

Supplementary Material

# Appendix B

All time-activity curves (TACs) of the muscle contours for all patients and scans are presented. TACs of the spheres were omitted, as they were highly similar across patients and did not provide additional value


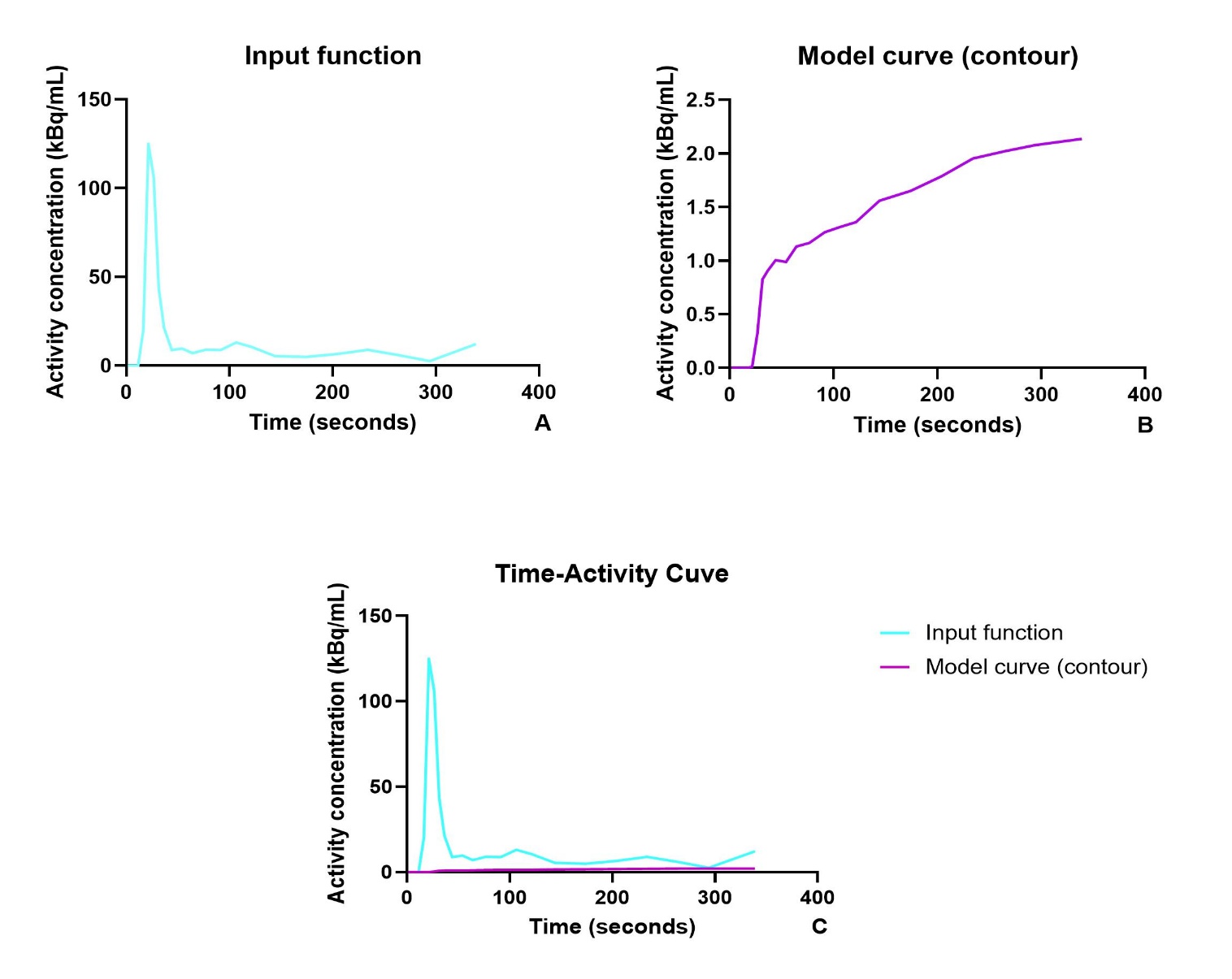


**Figure B1**. Left (affected) leg of first scan of the first patient. Time-activity curves for input function (A), derived from the superficial femoral artery, and (B) model curve, with the data points shown, derived from the muscle contour volume of interest. Colors correspond to the volumes of interest in images 1C and 3A.


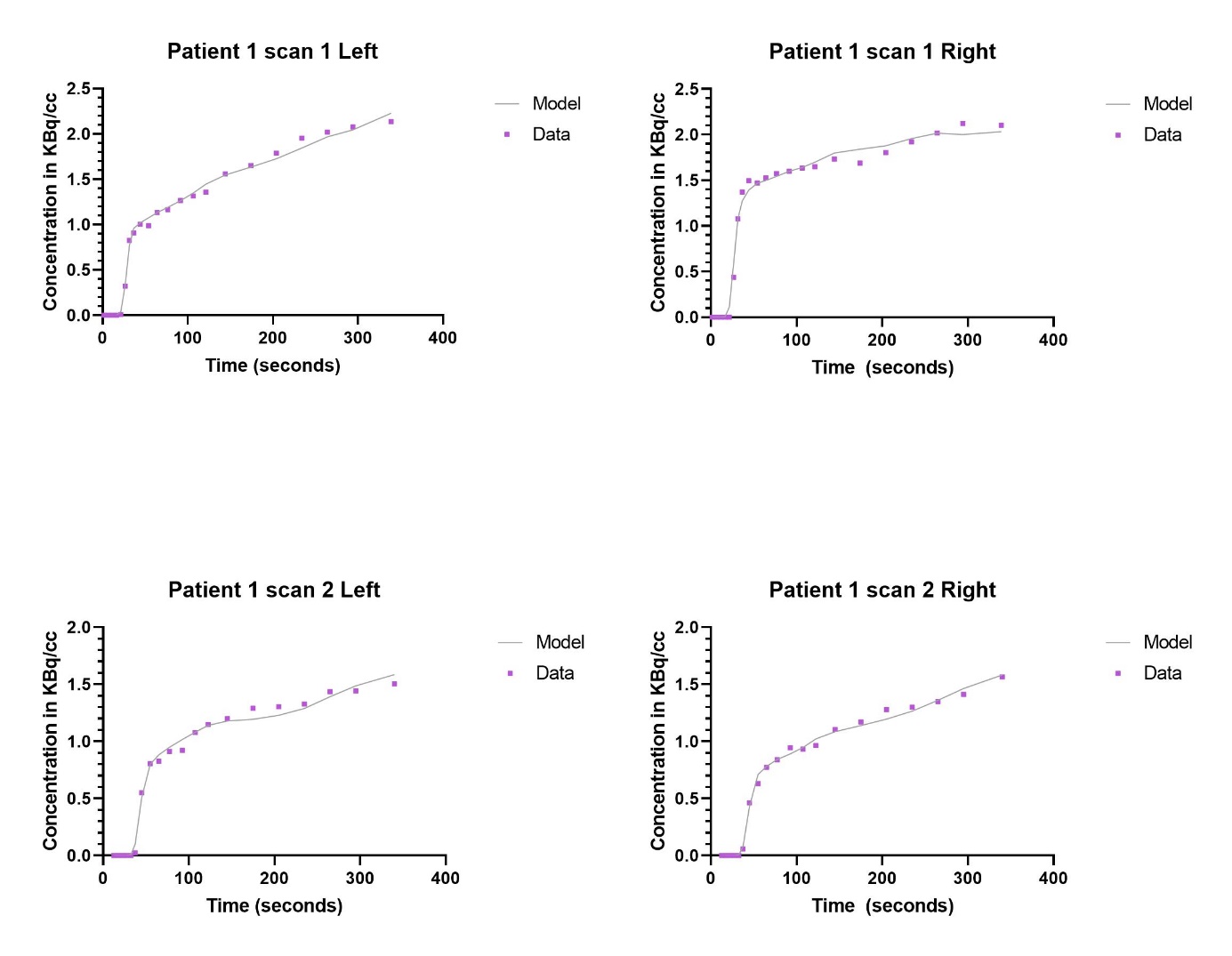


**Figure B2**. Patient 1. Time-activity curves for input function for two scans (above) and two legs. Colors correspond to the volumes of interest in image 4.


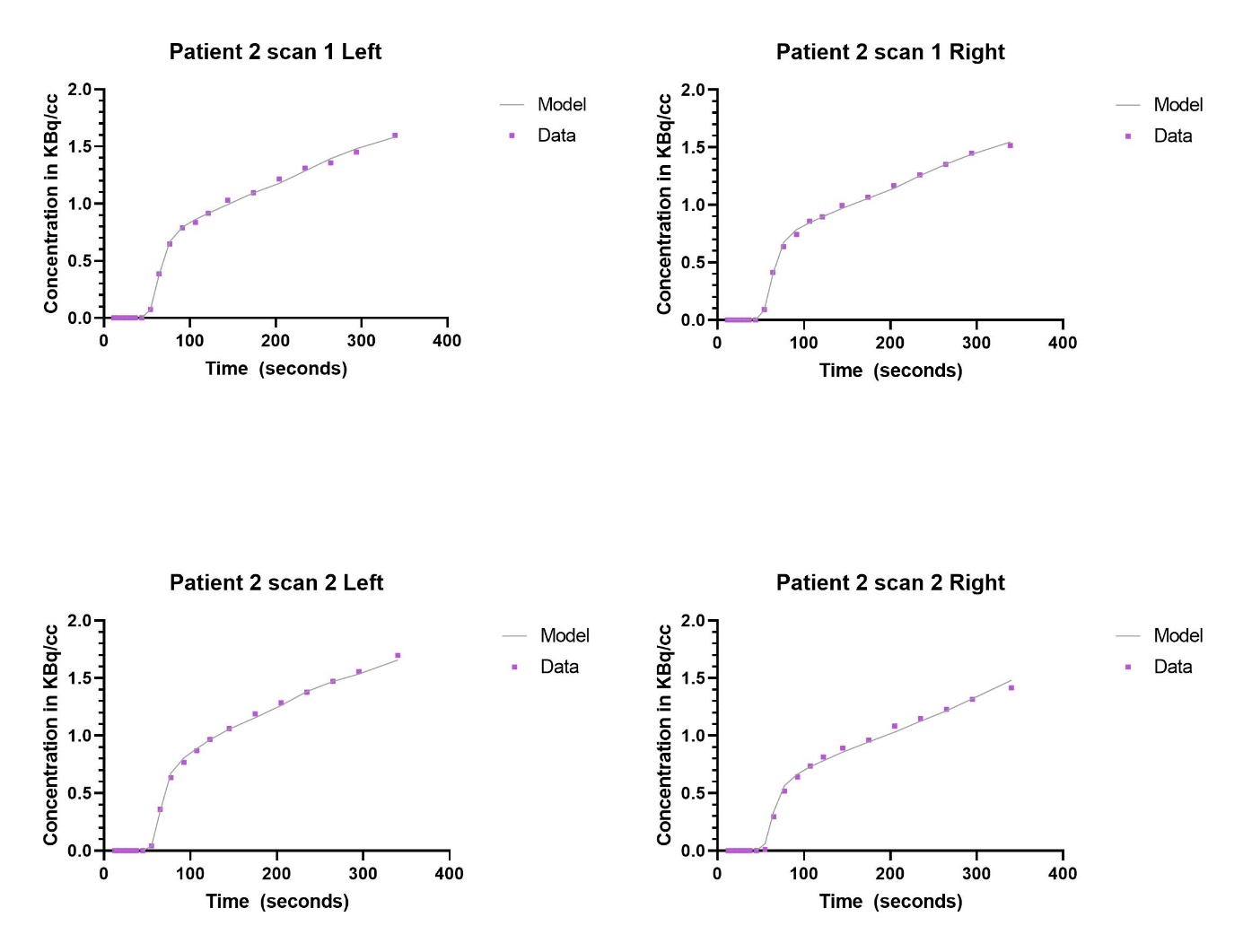


**Figure B3**. Patient 2. Time-activity curves for input function for two scans (above) and two legs. Colors correspond to the volumes of interest in image 4.


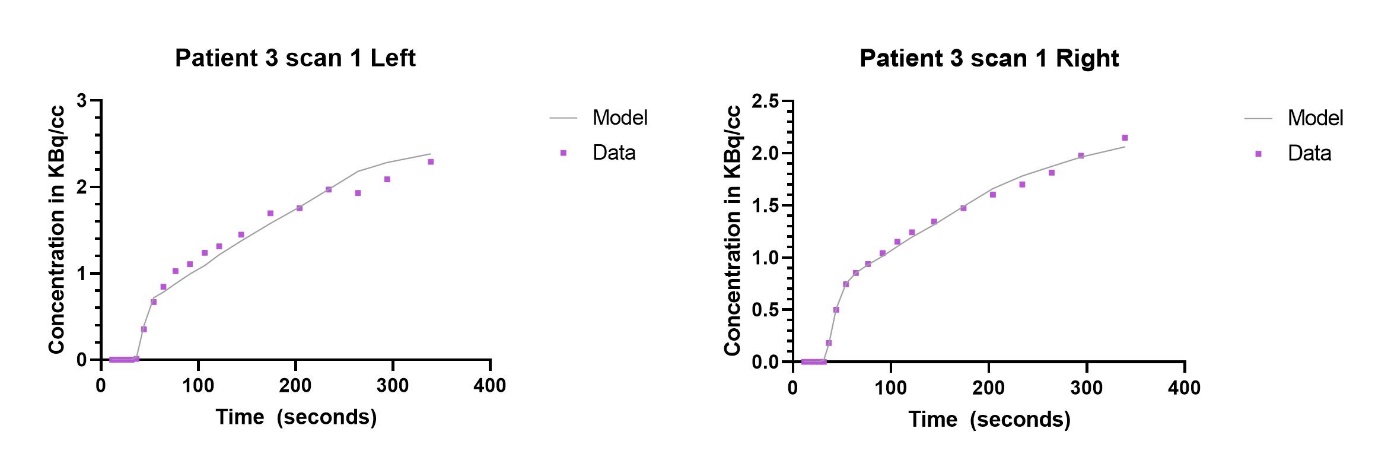


**Figure B4**. Patient 3. Time-activity curves for input function for two scans (above) and two legs. Colors correspond to the volumes of interest in image 4.
