# Supplementary material for: Feasibility of [15O]H2O PET-CT for quantifying lower limb muscle perfusion in peripheral arterial occlusive disease: a pilot study
Source: Front Nucl Med. 2026 Jan 2;5:1672054. doi: 10.3389/fnume.2025.1672054 (PMC12808382; doi:10.3389/fnume.2025.1672054)
Supplement: Supplementary file 3 [file Table3.docx]

Supplementary Material

# Appendix C

Bland-Altman plots for intrarater and interrater measurements.


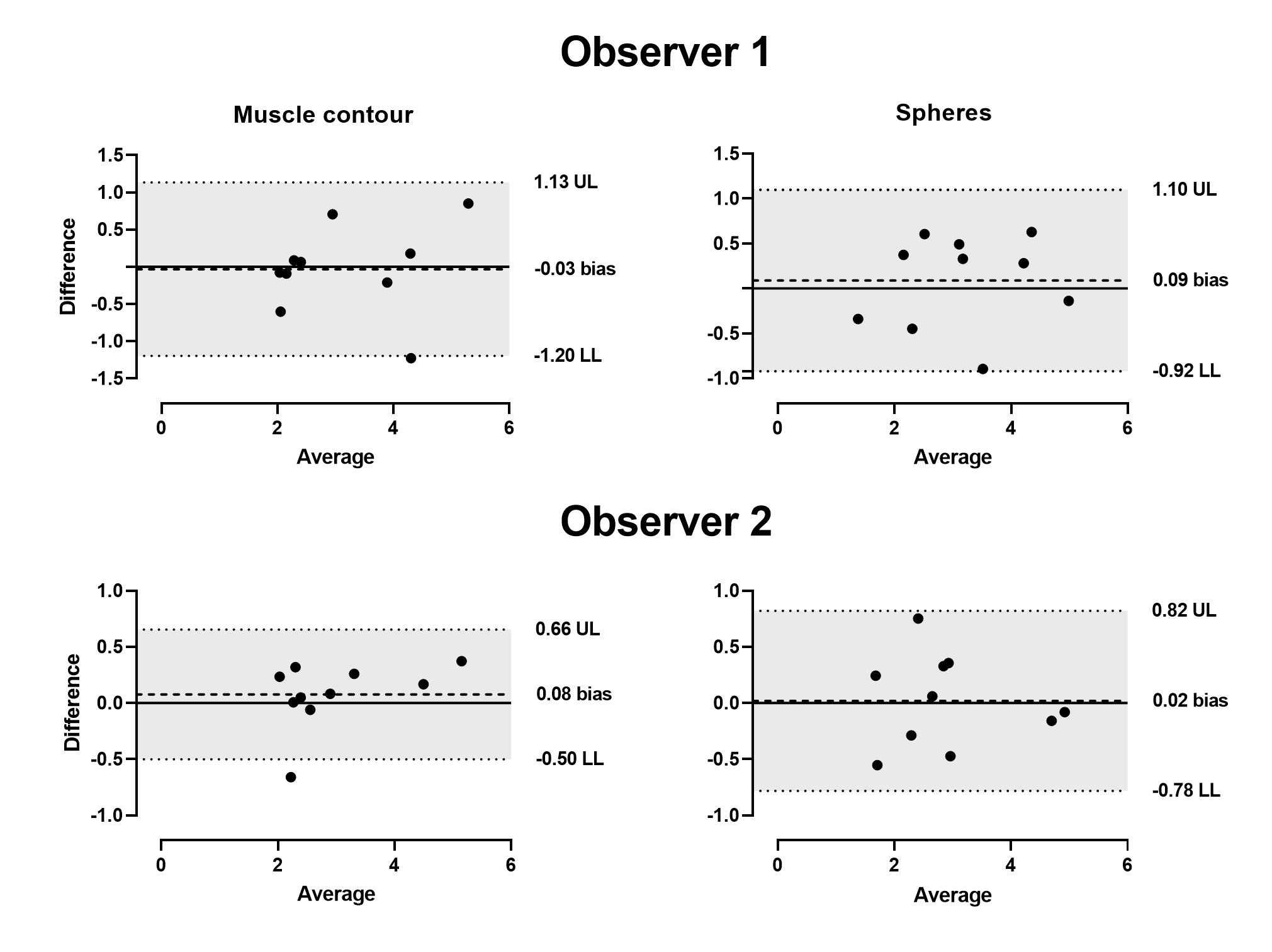


**Figure C1.** Bland-Altman plots of intrarater measurements for muscle contour (left) and sphere-based (right) volumes of interest for observer 1 and observer 2. The solid line represents the zero point, the dashed line represents the mean difference of activity concentration measurement, and the gray area between the dotted lines indicates the difference between the upper (UL) and lower limits (LL) of agreement. All values are expressed in mL/100 cm^3^/min.


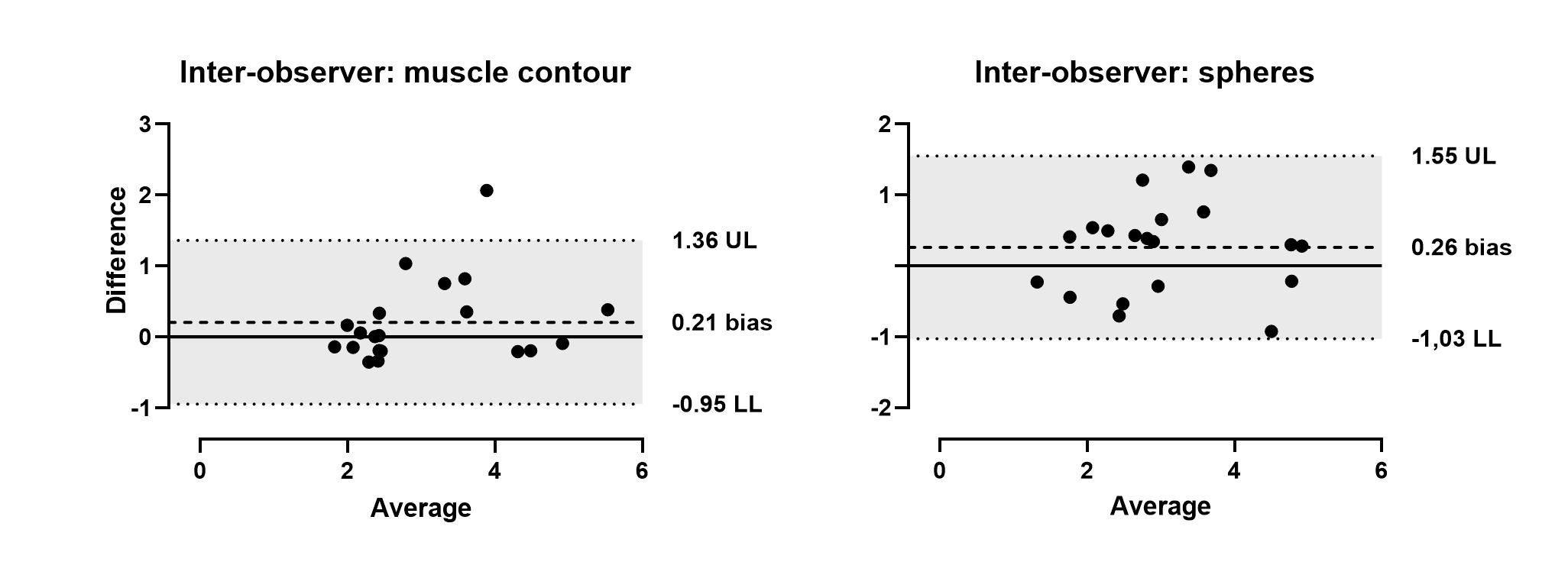


**Figure C2.** Bland-Altman plots of interrater measurements for muscle contour (left) and sphere-based (right) volumes of interest. The solid line represents the zero point, the dashed line represents the mean difference, and the gray area between the dotted lines indicates the difference between the upper (UL) and lower limits (LL) of agreement. All values are expressed in mL/100 cm^3^/min.
