# Supplementary figures and images for: Feasibility of [15O]H2O PET-CT for quantifying lower limb muscle perfusion in peripheral arterial occlusive disease: a pilot study
Source: Front Nucl Med. 2026 Jan 2;5:1672054. doi: 10.3389/fnume.2025.1672054 (PMC12808382; doi:10.3389/fnume.2025.1672054)

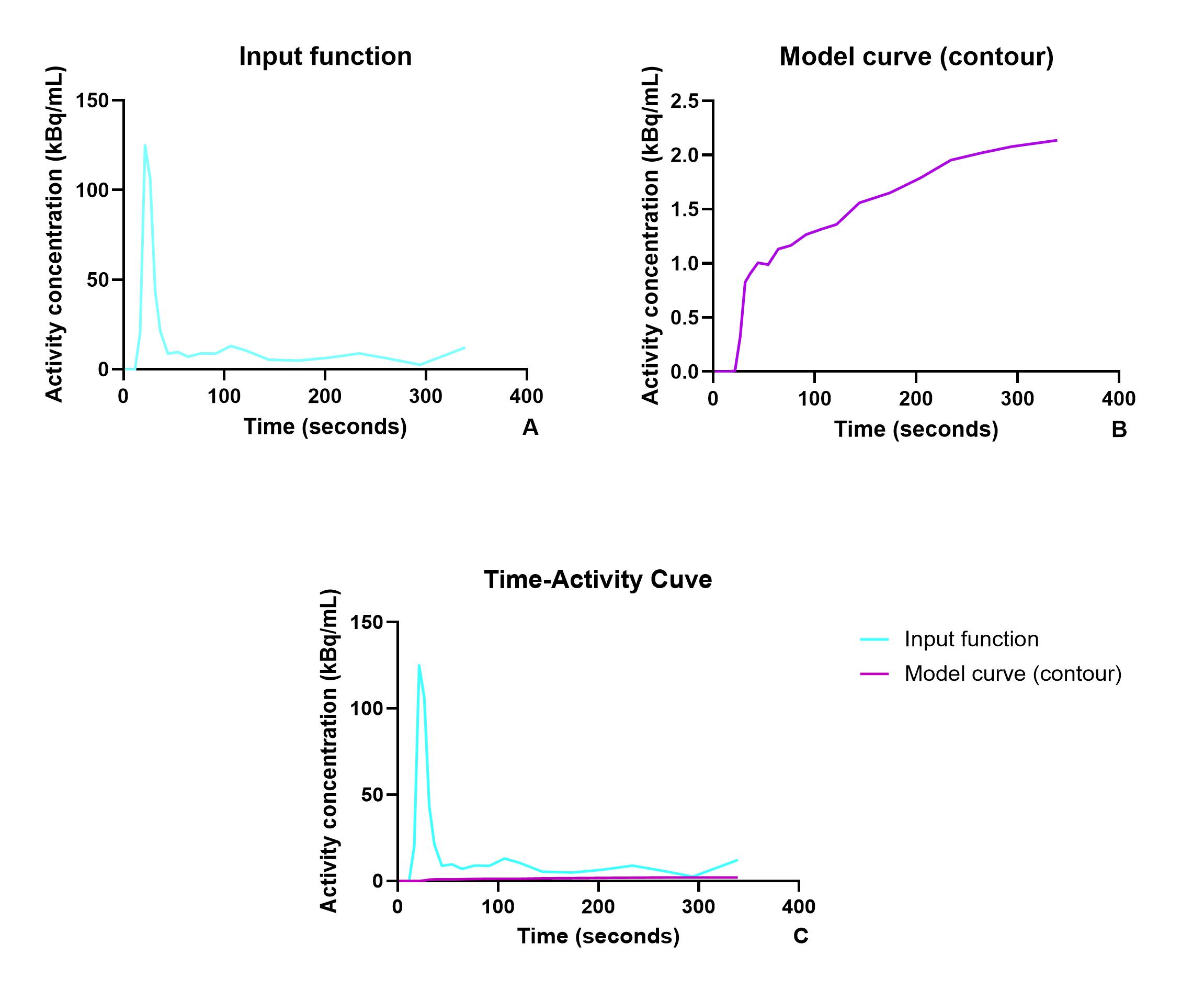

Supplement: Supplementary file 4 [file Image1.jpeg]

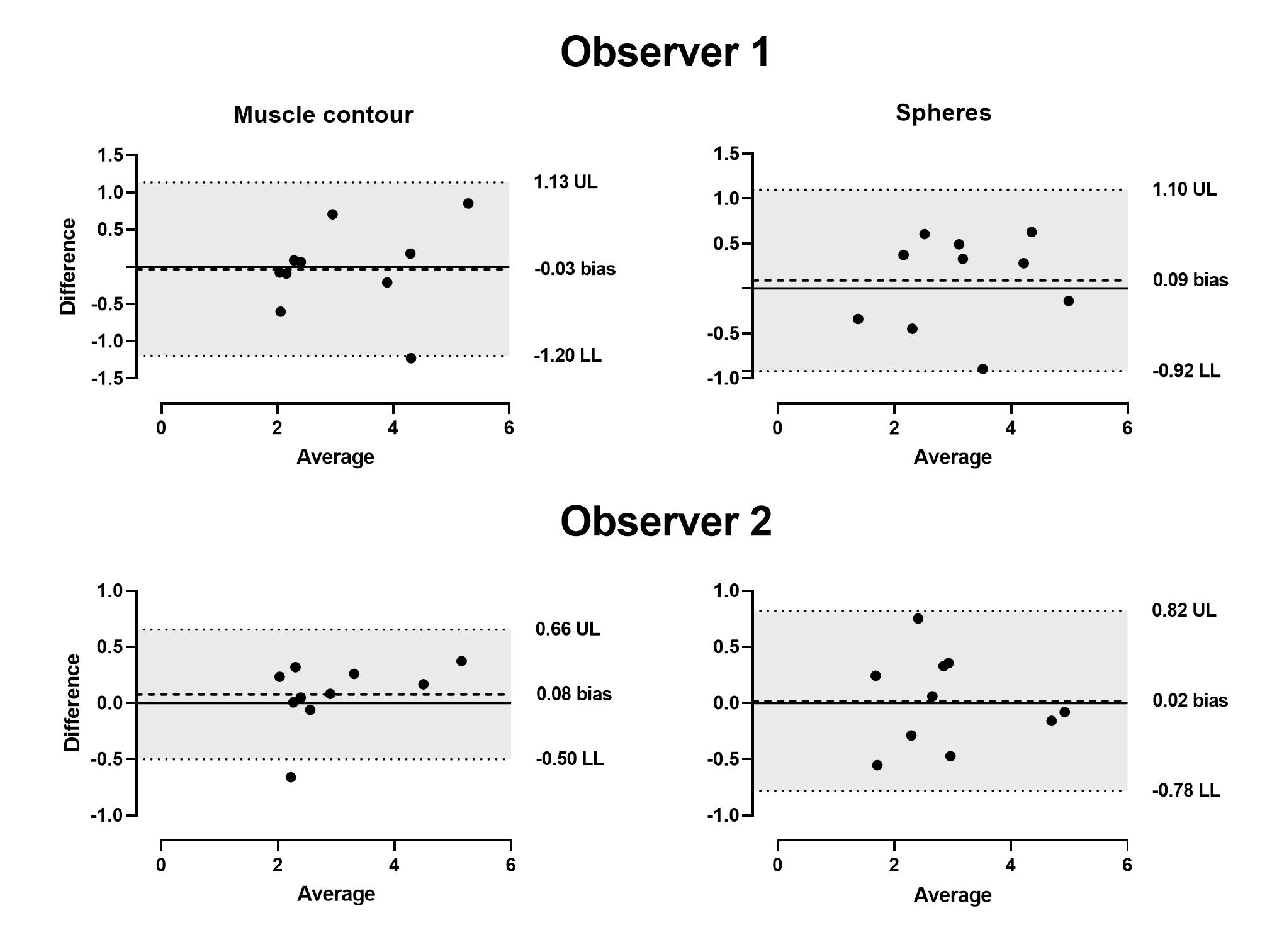

Supplement: Supplementary file 5 [file Image2.jpeg]

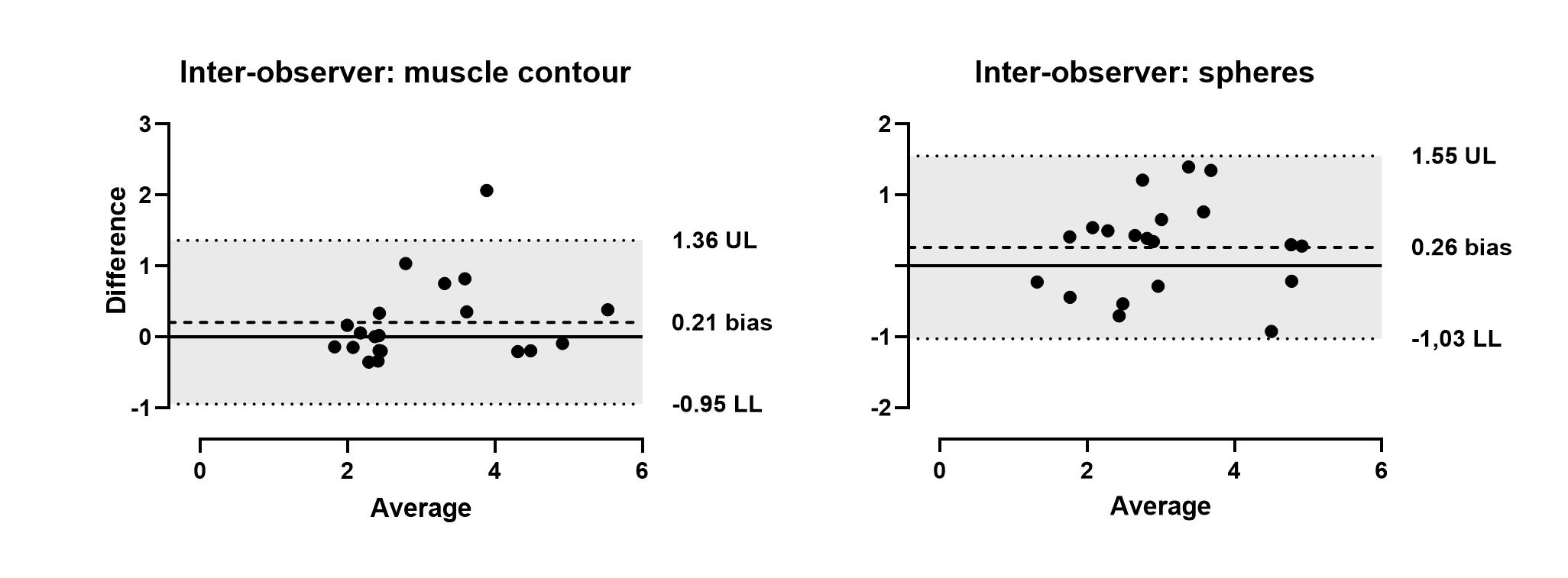

Supplement: Supplementary file 6 [file Image3.jpeg]
